# Electropositive Nanodiamond-Coated Quartz Microfiber Membranes for Virus and Dye Filtration

**DOI:** 10.1021/acsanm.1c00439

**Published:** 2021-03-09

**Authors:** Henry A. Bland, Isabella A. Centeleghe, Soumen Mandal, Evan L. H. Thomas, Jean-Yves Maillard, Oliver A. Williams

**Affiliations:** † School of Physics and Astronomy, Cardiff University, Queen’s Building, The Parade, Cardiff CF24 3AA, United Kingdom; ‡ School of Pharmacy and Pharmaceutical Sciences, Cardiff University, Redwood Building, King Edward VII Avenue, Cardiff CF10 3NB, United Kingdom

**Keywords:** nanodiamond, zeta potential, water filtration, virus filtration, electropositive membrane

## Abstract

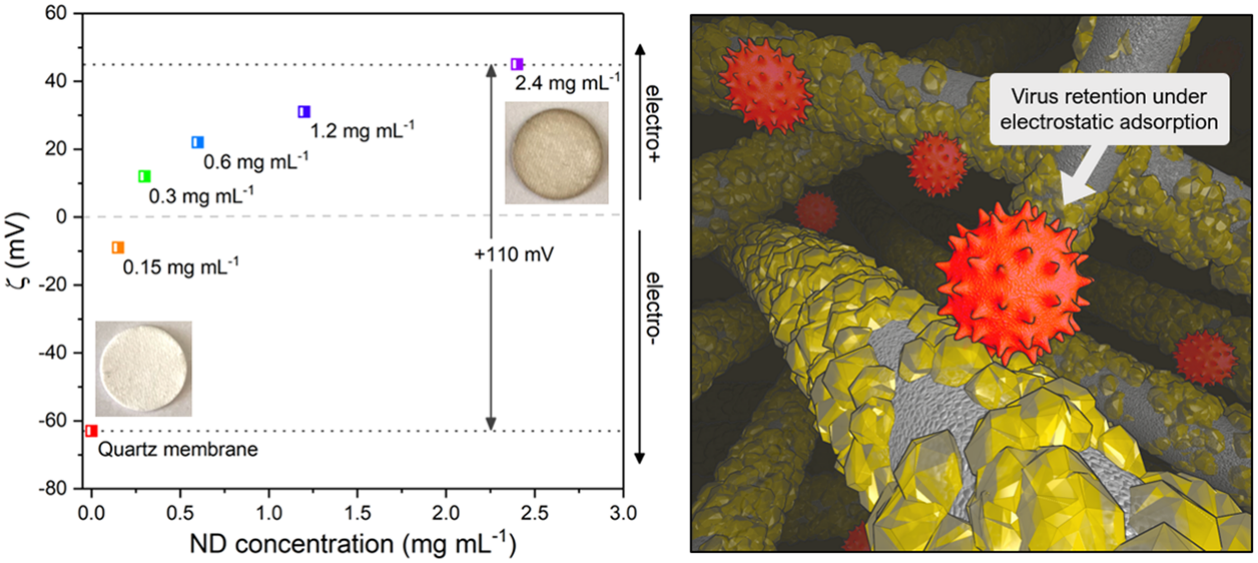

Electropositive membranes demonstrating high flux at low pressure differentials show great promise as universal separation platforms for viruses and other charged entities when centralized systems of water and power are scarce. However, the fabrication of a suitably stable membrane with optimal electrostatic characteristics remains a challenge. Here, hydrogenated detonation nanodiamond was loaded onto a quartz microfiber support membrane and coupled to the membrane surface under a high vacuum annealing process. The fabricated membranes display a zeta potential of +45 mV at pH 7 and an isoelectric point around pH 11. We show that the nanodiamond coating is robust to prolonged periods of pressurized water flow by performing extensive zeta potential measurements over time, and water filtration tests demonstrated excellent membrane retention for the electronegative dye molecule acid black 2, and at least a 6.2 log_10_ reduction in MS2 bacteriophage from feed waters (>99.9999%).

## Introduction

It is estimated that at least 1.2 billion people lack access to safe drinking water worldwide.^1,2^ Rapid population growth, urbanization, industrialization, and a changing climate continue to contribute to clean drinking water shortages for both developing and well-developed nations alike. One of the biggest challenges to universal clean drinking water is the presence of harmful nanoscale and subnanoscale contaminants, like bacteria, viruses, metals, metalloids, and the by-/waste- products of industry (pharmaceuticals, dyes, and pigments, etc.) that contaminate drinking water sources.^3−6^ While a number of separation platforms exist to target the removal of such contaminants, it is generally only with nanofiltration (NF) or reverse osmosis (RO) that high retention levels can been achieved.^7,8^ However, the relatively high costs of operation due to high power inputs and the requirement of large pressure differentials; the complexity of system design and maintenance; low chemical, mechanical, and/or thermal membrane stability; and an extreme susceptibility to fouling, have historically limited their widespread use to more centralized water treatment systems.^9−11^

In recent years adsorptive depth filtration (ADF) has emerged as a promising method to control nanocontaminant levels in drinking water. ADF targets the removal of contaminants under the influence of van der Waals forces, electrostatic forces, and/or hydrophobic interactions, with long-range electrostatic forces primarily driving contaminant adsorption to the membrane surface. Since retention is not achieved through size exclusion, as in NF/RO, ADF membranes may be fabricated to encompass a greater average pore size, and hence smaller pressure differentials are required to generate water flow. Consequently, ADF membranes can operate at a fraction of the running cost and with little to no electrical input, making them tremendously attractive as a universal method of water purification capable of operation in the most extreme and remote of environments.

While van der Waals forces are always attractive, electrostatic forces can be attractive or repulsive depending on the relative charge of the membrane surface and the target contaminant. The electronegative surface of traditional membranes, particularly silicon-based ceramics like glass, quartz, and diatomaceous earths, make direct adsorption difficult at typical pH values observed for water, since the vast majority of problem contaminants exhibit a similarly negative charge.^12−16^ Electropositive coatings have thus been applied to the membrane surface that allow for the retention of negatively charged contaminants under electrostatic adsorption. Polyelectrolytes,^17,18^ metallic salts,^19,20^ zirconia,^21,22^ yttria,^15,23−25^ copper oxides,^26^ hematite,^27^ magnesia,^28,29^ alumina,^30^ and amino-silanized yttria-stabilized zirconia^31^ are among those materials more recently explored. However, electropositive coatings such as these have typically suffered from poor adhesion to their membrane support, low isoelectric points (p*I*), low overall zeta potentials, and the use of potentially toxic materials. Since the magnitude of the retention achieved by an ADF membrane is proportional to the magnitude of the surface charge of the membrane over prolonged periods of water filtration, the importance of the magnitude of the zeta potential and durability of the coating cannot be overstated.

Diamond boasts many superlative properties including extreme hardness, good biocompatibility, and a highly functionizable surface.^32−35^ It has found application across microbiology and medicine, as imaging and sensing agents, in drug delivery, and for its promising antibacterial properties.^36−39^ When detonation nanodiamond is annealed under a hydrogen atmosphere, the nanodiamond surface may be granted a zeta potential upward of +60 mV at pH 7 and an isoelectric point greater than pH 12, some of the highest values reported in the literature.^40,41^ However, in order to exploit the extreme electropositive charge density exhibited by hydrogenated detonation nanodiamond, it must first be incorporated into a system, or a component thereof, suitable for long-term water filtration. The principle focus of this work, therefore, was to design a fabrication pathway whereby nanodiamond could be loaded and coupled to a membrane support structure.^42^ The retention capabilities of the fabricated membrane would then be evaluated for the organic dye molecule acid black 2 and the bacterial virus MS2.

## Results and Discussion

### Membrane Fabrication

**Figure 1:**
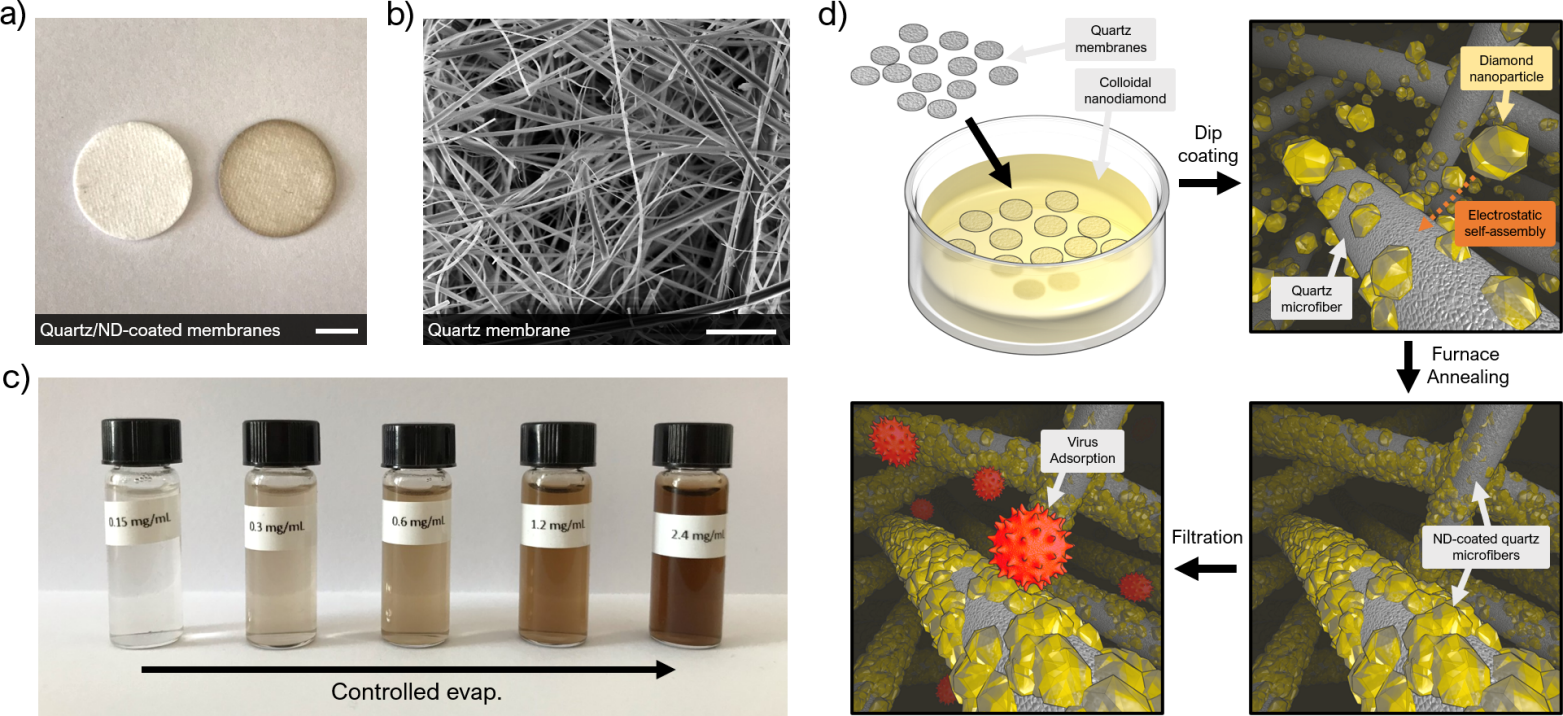
Fabrication of ND-coated quartz microfiber membranes. (a) A photograph of an uncoated quartz membrane (left) and a ND-coated quartz membrane (right). The scale bar is 5 mm. (b) An SEM micrograph of the uncoated quartz membrane. The scale bar is 10 μm. (c) A photograph of stable ND colloids produced by controlled evaporations. ND concentration increases from left to right, as labeled. (d) A schematic showing the stages ofmembrane fabrication, including the dip-coating process, the self-assembly of ND on the quartz support membrane, and membrane annealing.

A commercially available quartz fiber depth filter membrane, type AQFA, was selected as a support structure upon which the electropositive nanodiamond would be loaded. In general, ceramic membranes have been favored for their high mechanical strength and chemical stability.^43,44^ The quartz membrane is shown in Figure 1a (left). The membrane is 430 μm thick and cut to 14 mm discs. A scanning electron microscope (SEM) micrograph of the membrane is displayed in Figure 1b; it exhibits a mesh of quartz fibers primarily in the submicron range. The open, fibrous structure was selected to provide high water flux at low pressure differentials; a water flux of 960 L m^–2^ h^–1^ is stated by the manufacturer; however, the associated pressure is not stipulated.

**Figure 2:**
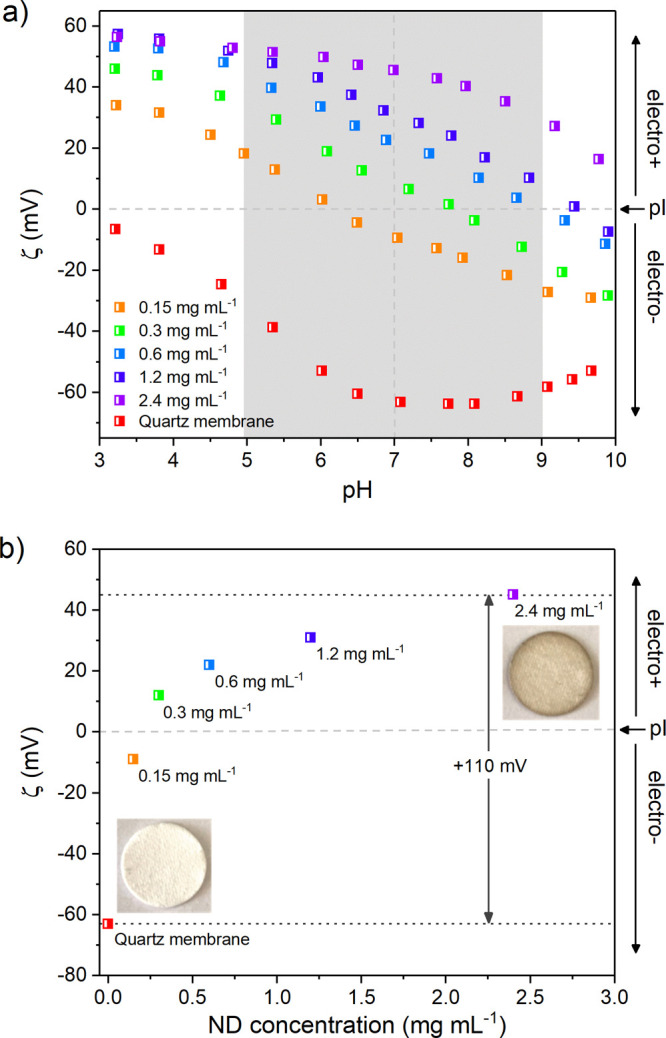
Zeta potential measurements for ND-coated quartz microfiber membranes. (a) Zeta potential versus pH for membranes dip-coated with various ND concentrations. (b) Membrane zeta potential at pH 7 versus concentration of ND colloid used in fabrication. The maximum shift in zeta potential of +110 mV is marked. Inset are images of the uncoated and ND-coated membranes.

Quartz microfiber membranes were dip-coated in a bath of colloidal hydrogenated detonation nanodiamond (ND), produced by techniques described in the Materials and Methods section.^40^ The dip-coating process is shown in the diagrams of Figure 1d. The ND colloid saturates the quartz membrane totally and adheres to the microfiber surface principally under electrostatic forces of attraction that exist between the electropositive ND and the electronegative membrane (described in more detail in Figure 2).^45^ To achieve greater ND coating densities, the concentration of colloidal ND was enhanced by gentle evaporation of water from the colloid prior to membrane dip-coating. A photograph of colloidal ND samples used in fabrication is shown in Figure 1c. By carrying out controlled evaporations, concentrations of ND up to 2.4 mg mL^–1^ were achieved: a 16-fold increase from the initial concentration of 0.15 mg mL^–1^. 2.4 mg mL^–1^ emerged as the concentration limit within the scope of these experiments, as thereafter the colloidal stability began to collapse, and large ND aggregates formed. A photograph of a ND-coated membrane is shown in Figure 1a (right). During the final stages of fabrication, the ND-coated membranes were annealed under high temperature and vacuum, prompting the formation of a strong bonding interaction between the ND coating and the quartz surface, thereby integrating ND into the membrane structure. The membranes were then rigorously washed to remove any unbound ND.

### Membrane Zeta Potential

A reliable method for determining a material’s surface charge is crucial for the fabrication of a membrane proposed to exhibit extreme electropositive characteristics. Typically, the zeta potential (ζ), defined as the potential difference between the stationary layer of fluid at a material’s surface and the bulk aqueous phase that surrounds it, is measured through electrokinetic effects, and presented as the best possible experimental indicator of surface charge.

Zeta potential measurements for the uncoated quartz membrane and membranes dip-coated in colloidal ND of increasing concentration are presented in Figure 2a, as a function of pH. The uncoated membrane shows a strong negative zeta potential across the measured pH range; the data is extrapolated at the lower end of the pH scale and we predict the isoelectric point (pI) to be somewhere in the region of pH 2.5. The result correlates reasonably well with the published literature on quartz (although differences in the measurement technique provide a level of disparity). The negative zeta potential in this case is attributed to the deprotonation of oxygen-based functional groups, such as alcohols, at the quartz surface.^46^ Once dip-coated in colloidal ND, the membrane zeta potential experiences a shift toward the positive, as the electronegative surface groups of the quartz are shielded by the electropositive ND. More concentrated ND colloids drive self-assembly of ND at the membrane surface, and the zeta potential is pushed further into the positive. The ND-coated membrane produced by coating with a 2.4 mg mL^–1^ ND colloid shows a switch in zeta potential of +110 mV at pH 7, as displayed in Figure 2b, and produces a highly electropositive membrane, which persists across the entire pH range. At pH 7, it displays an overall zeta potential of +45 mV; to our knowledge, it is by a considerable margin the most electropositive membrane reported in the literature. The zeta potential eventually decays with increasing electrolyte pH, and we predict the pI to be somewhere in the region of pH 11.

How the membrane will react to real-world conditions must also be considered here, since such conditions will often differ from those used to measure zeta potential. Surface and ground water may possess a high salt content that will act to reduce the Debye length of the membrane surface, the distance to which the electrical potential of the membrane surface persists into the surrounding medium, thereby reducing the membrane’s overall efficiency and capacity. The magnitude of the electropositive surface character is therefore critical in order to maximize the Debye length and therefore the membrane’s retention capabilites, and highlights again the significance of the high overall zeta potential exhibited by the ND-coated membrane.

As a preliminary indicator of suitability for virus filtration, the shaded region in Figure 2a signifies the pH range over which a system must remove 99.99% of viruses from feedwater containing 10^7^ plaque forming units per litre (pfu/L), so as to be considered a true virus filter by the United States Environmental Protection Agency (USEPA).^23^ Since virus p*I*’s typically range between 3.5 and 7,^14^ the highly positive zeta potential exhibited by the ND-coated membrane over such a wide pH range is predicted to provide good virus retention under the influence of electrostatic adsorption alone. Given optimal water conditions, virus retention by the membrane should be possible up to the membrane’s p*I* at around pH 11, and since the magnitude of the retention that can be achieved by a membrane acting under electrostatic interactions is proportional to the magnitude of membrane zeta potential, high retention levels are anticipated.

### Membrane Morphology and Composition

**Figure 3:**
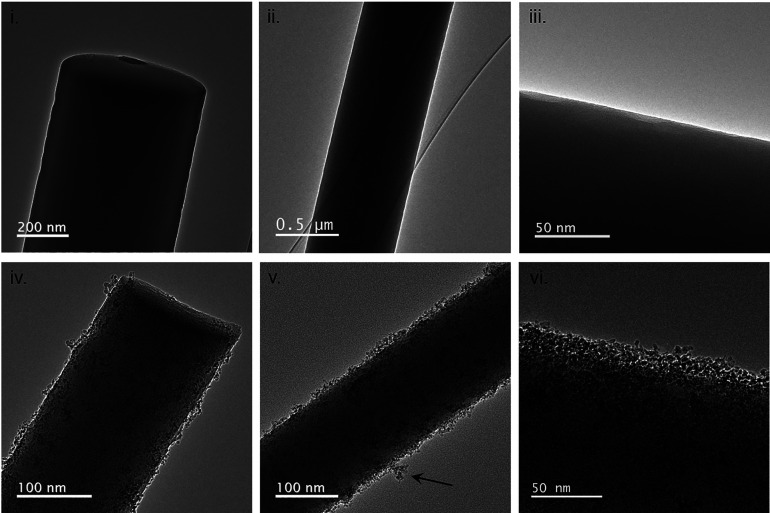
ND-coated quartz microfiber membrane morphology and composition. Bright field transmission electron microscope micrographs displaying microfiber morphology of the uncoated quartz microfibers (i–iii) and ND-coated quartz microfibers (iv−vi). The dipcoating concentration is 2.4 mg mL^–1^.

Membrane surface morphology is evaluated in the transmission electron microscope (TEM) micrographs of Figure 3. Membranes were broken open and individual fibers examined to determine the extent and nature of the coating. Figure 3i–iii show uncoated quartz microfibers. The fiber surface is uninterrupted and smooth. Figure 3iv–vi show quartz microfibers taken from membranes dip-coated in a 2.4 mg mL^–1^ ND colloid, and display a quartz surface covered in tightly packed ND. Marked by the black arrow in Figure 3v, ND aggregates have formed at the fiber surface that were not removed by the final washing stages of fabrication. Aggregates would tend to suggest that loading mechanisms beyond electrostatic self-assembly may take over once a ND monolayer has formed, since the electronegative quartz surface driving self-assembly is utterly shielded from colloidal ND thereafter. A fraction of the ND coating must therefore be attributed to interactions involving the packing of ND, due to its irregular shape and/or due to localized areas of low zeta potential where electrostatic repulsion is overcome by ND particle velocity during fabrication, and that allows for ND particle collisions where close-range van der Waals attractive forces dominate.

**Figure 4:**
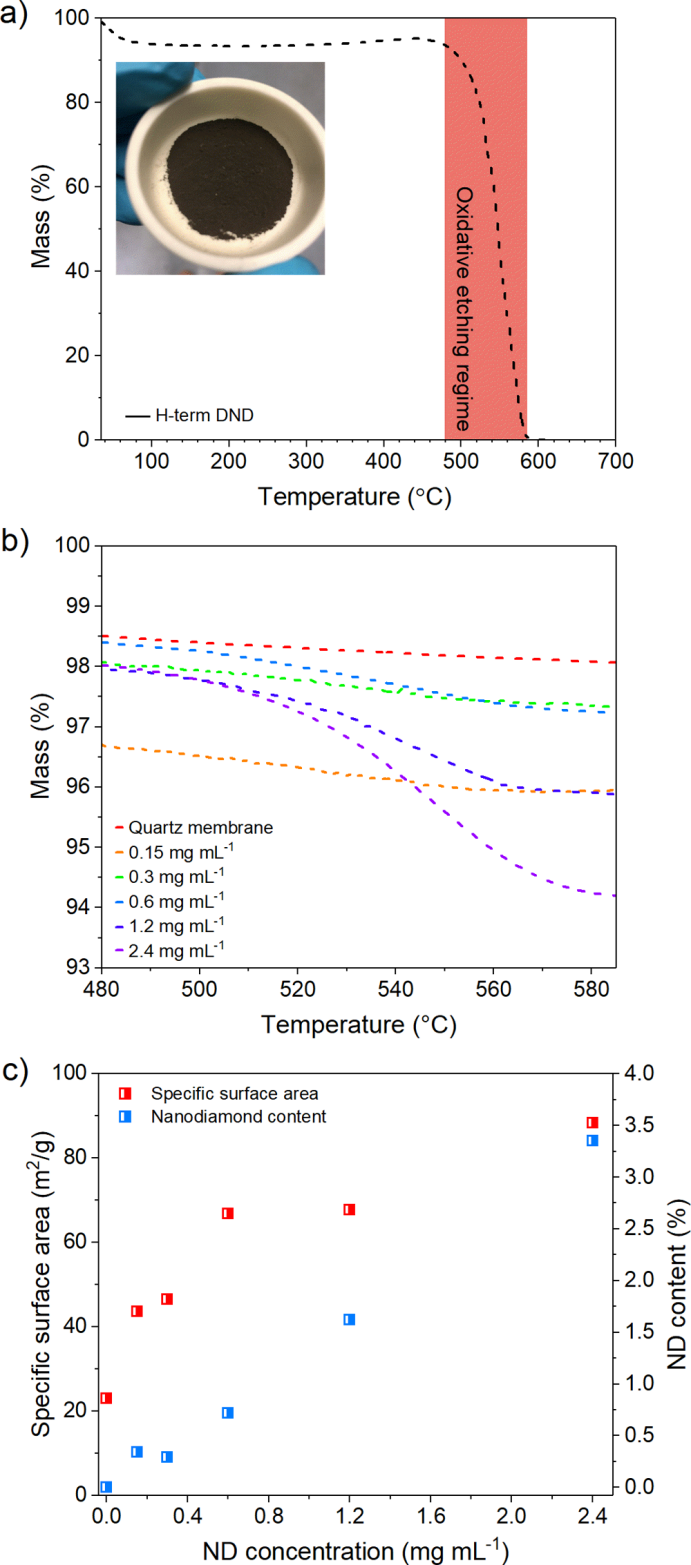
(a) Thermogravimetric analysis of hydrogenated detonation nanodiamond. The shaded region indicates the temperature range of the oxidative etch. Inset is an image of a ND powder sample. (b) Thermogravimetric analysis of each membrane for the temperature range of the oxidative etch. (c) Nanodiamond content and BET specific surface area of the membranes as a function of the concentration of ND colloid used in fabrication.

Quantitative analyses of the ND coating were achieved by thermogravimetric analysis (TGA) and BET specific surface area analysis; the results are presented in Figure 4a–c. During TGA, we take advantage of the disparity in thermal stabilities between ND and quartz to determine the mass of nanodiamond present on the fabricated membranes. First, pure ND powder was heated in air, between 30 and 900 °C to determine the temperature range over which it would be oxidatively etched. The plot shown in Figure 4a displays the TGA curve for ND, and the etch temperature range between 480 and 585 °C is marked. The etch range was defined as the temperature range between the point at which the sample mass dropped below the original mass (following water desorption at 100 °C) and the point at which the sample mass dropped below 1% of its original mass (more information may be found in the Methods and Materials section). TGA curves derived from the untreated membrane and all coating concentrations of ND-coated membranes were then analyzed across the same temperature range, and results are shown in Figure 4b. The percentage decrease in sample mass equivalent to the mass of ND loaded onto each membrane is shown in Figure 4c. Membrane ND content scaled linearly with the concentration of the ND colloid used in its fabrication. The 2.4 mg mL^–1^ ND colloid produced the most heavily coated membrane and was comprised of approximately 3.4% ND by mass. The linear coating trend displayed here confirms the persistence of semistable aggregates on the microfiber surface following fabrication and washing processes.

Each membrane is 430 μm thick, cut to 14 mm discs, and weighs approximately 13.3 mg. Assuming a 3.4% coating is achieved using a 2.4 mg mL^−1^ nanodiamond colloid, each coated membrane encompasses around 0.45 mg of hydrogenated detonation nanodiamond.

Since adsorption under electrostatic forces is a surface effect, the magnitude of the retention achieved by such a separation platform is proportional to the magnitude of both the surface charge and the surface area of the electrostatically active membrane. BET specific surface area analysis is shown in Figure 4c, as a function of the colloidal ND concentration used in the fabrication. The uncoated membrane displays a specific surface area of 23 m^2^/g. The ND-coated membrane (3.4% ND) experiences a near 4-fold increase in specific surface area to 88 m^2^/g upon dip-coating. The data again display a somewhat linear relationship with regard to increasing colloidal ND concentration, although the trend in this case is far less linear, and to what extent the aggregates contribute to the surface area, and ultimately to filtration, is unknown. Nevertheless, it is evident that the coating significantly increases the surface area of the membrane above the uncoated level.

### Stability of the Nanodiamond Coating

The nature of the bonding interaction between the ND coating and the quartz surface was exceptionally difficult to characterize by spectroscopic techniques, primarily due to the small number of atoms contributing to the bonding interaction itself. Its position around a cylindrical support structure and the packing density of 5 nm particles of irregular shape made imaging the interface similarly problematic. Instead, focus was placed on determining whether the coating was robust under pressurized water flow, such as that which may be used when the membrane is in operation.

**Figure 5:**
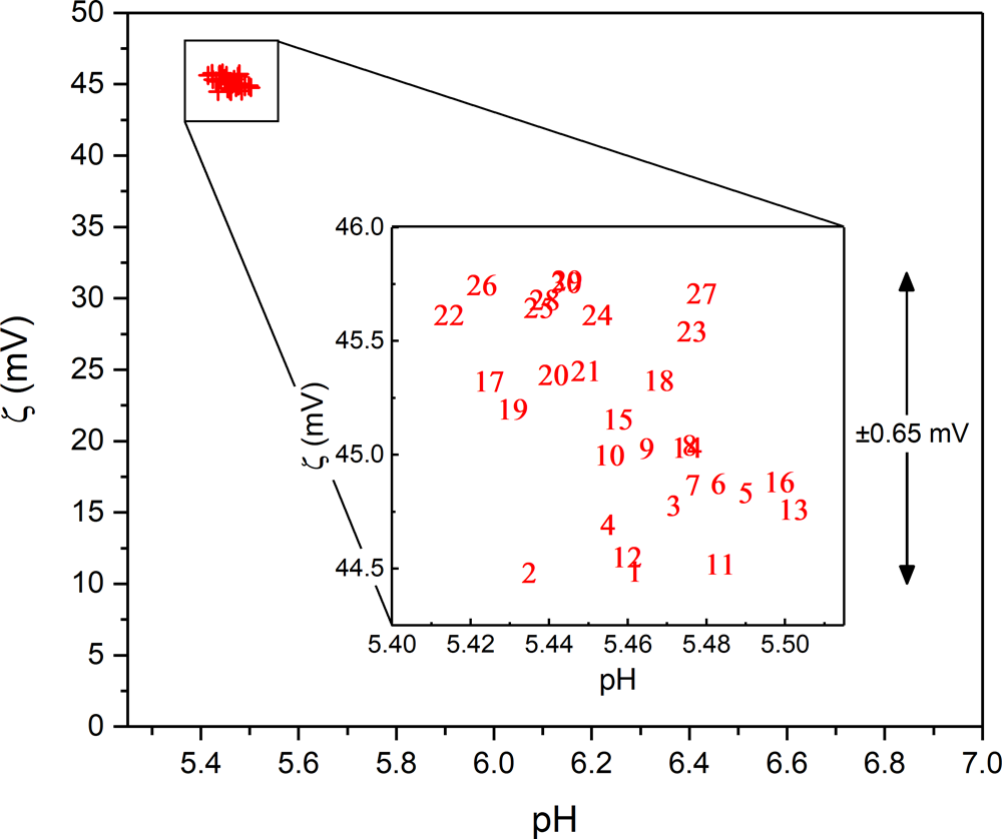
Nanodiamond coating stability. Repeat zeta potential measurements for ND-coated membranes (3.4% ND) at pH 5.5. Inset is a magnified view of the cluster of data points, where each data point has been numbered in the sequence that it was taken.

In order to replicate filtration conditions, the “permeation mode” set up of the SurPASS 3 electrokinetic analyzer is exploited. The system is in effect a small-scale model of the membrane in operation. For each zeta potential measurement, 200 mL of electrolyte solution passes through ∼0.2 cm^3^ of the membrane under mechanically applied water pressure between 200 and 600 mbar. Successive zeta potential measurements were taken and any changes to the membrane zeta potential between each run were identified. Since membrane zeta potential is dependent upon the surface coverage of the coating, any nanodiamond dissociation below a single monolayer from the membrane support would be revealed as a decrease in potential. Thirty successive zeta potential measurements were taken of the nanodiamond-coated membrane (3.4% ND), for the same pH value, and the results are plotted in Figure 5. The red cross data points are all closely grouped around +45 mV and have a total range ∼1.3 mV. The inset plot is a magnified view of the cluster where each of the 30 zeta measurements has been numbered in the sequence that it was taken. The inset plot displays no discernible decreasing trend between successive zeta potential measurements, and so within the parameters of the experiment, the coating is preserved under water flow to at least to a single nanodiamond monolayer.

### Retention Performance

**Figure 6:**
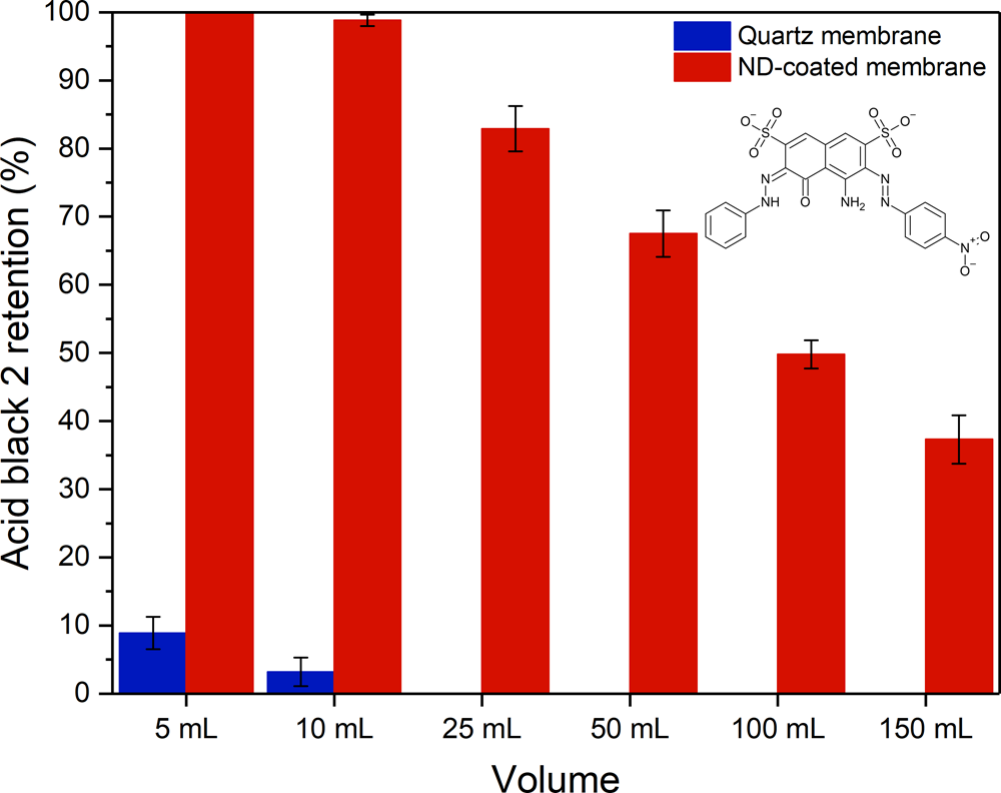
Retention performance versus acid black 2. Retention performance of uncoated and ND-coated membrane (3.4% ND) stacks for the dye molecule acid black 2 in deionized water (10 mg/L). The error bars are ± the standard deviation calculated for three repeat measurements, using a fresh membrane stack for each repeat.

Performance of the membrane was first evaluated for the organic dye acid black 2, an electronegative molecule (molecular weight 616.49) used in the dying of leathers, woods, and textiles and in the manufacture of inks. The occurrence of organic dyes in drinking water has become increasingly more widespread in many countries, in the face of growing industrialization. Since synthetic dye molecules are so small, on the scale of a single nanometer or less, they are an excellent test of the membrane’s retention performance based on electrostatic adsorption alone. The chemical structure of acid black 2 is inset in Figure 6.

**Figure 7:**
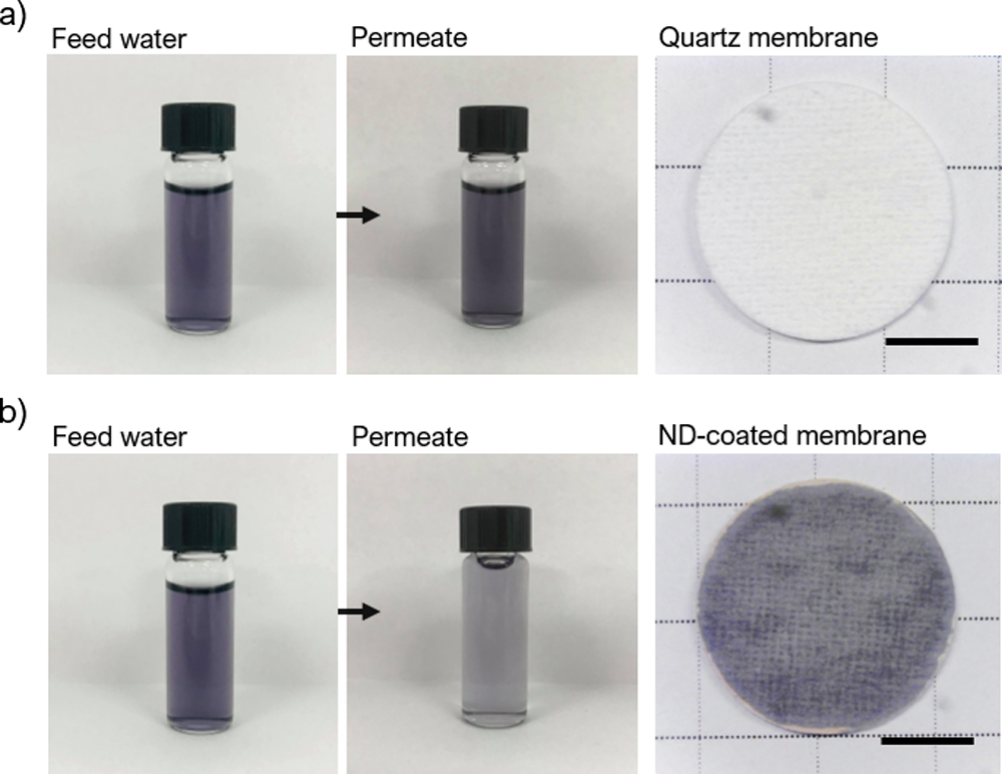
(a) Photographs of sample feed water and permeate after 150 mL of the feed water is passed through an uncoated membrane stack (left). A single uncoated membrane taken from the membrane stack following the retention test (right). The scale bar is 5 mm. (b) Photographs of sample feed water and permeate after 150 mL of the feed water is passed through a ND-coated membrane stack (3.4% ND) (left). A single ND-coated membrane taken from the membrane stack following the retention test (right). The scale bar is 5 mm.

Acid black 2 was dissolved in deionized water at pH 7 and made up to a concentration of 10 mg/L. A total of 150 mL of the acid black 2 containing feed water was flowed through stacks of ten uncoated and ND-coated membrane discs (14 mm diameter, 4.3 mm total thickness, 0.66 cm^3^ total volume) under applied pressures between 0.25 and 0.5 bar, and the results are plotted in Figure 6. To determine membrane retention (*R*), the absorbance of the dye molecule in the feed water (Abs_f_) and the permeate (Abs_p_) was evaluated at the relevant absorption maximum at 575 nm, using an ultraviolet–visible spectrometer (see eq 1).
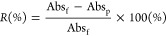
1Permeate samples were analyzed at set volumetric intervals in order to consider any changes to retention performance over large volumes of processed water. The uncoated membrane showed very little retention for the dye molecule, less than 10% for 5 and 10 mL of processed feed water, and 0% retention thereafter. Photographs of feedwater and permeate samples taken after 150 mL was passed through the uncoated membrane stack are shown on the left side in Figure 7a, and a single uncoated membrane taken from the membrane stack after the retention test is shown on the right side in Figure 7a. The membrane itself shows no signs of dye molecule retention. The similar charges on both the acid black 2 molecule and the quartz membrane are expected to produce an electrostatic repulsive force between the two, preventing the dye molecule from approaching the membrane surface where close-range van der Waals attractive forces could promote adsorption. The ND-coated membranes (3.4% ND) show high levels of retention for the dye molecule; more than a 90% greater retention at lower filtered volumes when compared to the uncoated membrane alone. Photographs of sample feedwater and permeate after 150 mL of feed water is passed through a ND-coated membrane stack are shown in the photographs on the left side of Figure 7b, and a single ND-coated membrane taken from the membrane stack after the retention test is shown on the right side of Figure 7b. The membrane exhibits clear discoloration due to dye molecule retention. The substantial difference in retention between the two membranes can plainly be attributed to adsorption based on the electrostatic interactions of the dye molecule and the ND-coated membrane (see Figure 2a), since any sieving effects are extremely unlikely, although contributions from other sorption mechanisms at the nanodiamond surface cannot be ruled out entirely. Typical for adsorption-based filtration, retention performance declines over time as more of the membrane surface is coated by the adsorbate. The electric potential of the membrane surface tends toward an equilibrium state as it becomes electrostatically balanced by the counter charges on the acid black 2 molecule. As localized sites of high zeta potential become ever more diffuse, the probability of further adsorption across the membrane declines.

**Figure 8:**
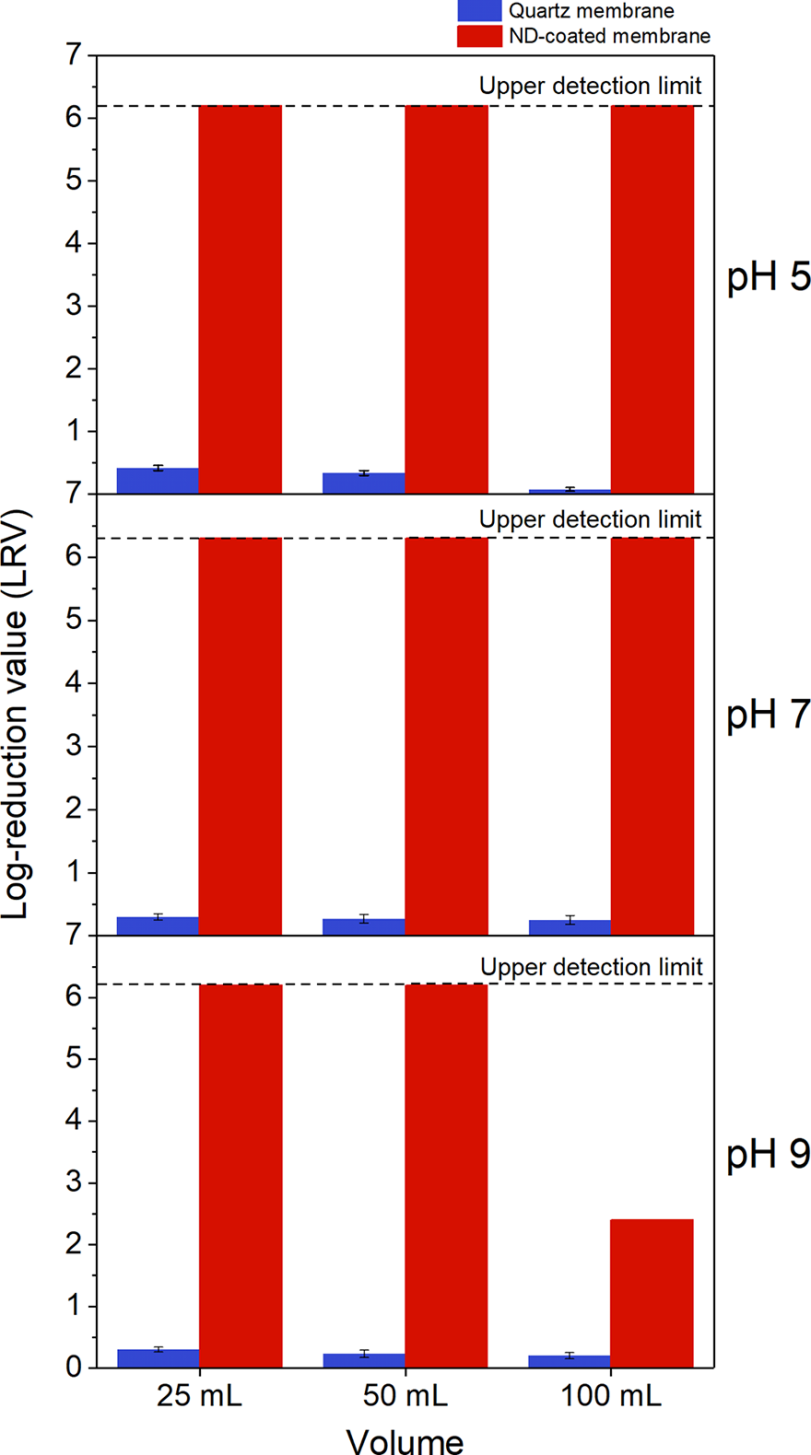
Retention performance versus bacteriophage MS2. Retention performance of stacks of uncoated and ND-coated membranes for the bacteriophage MS2 (10^7^ pfu/mL) across three pH values. Error bars are ± the standard deviation calculated for three independent biological replicates. MS2 bacteriophage retention by the ND-coated membrane was shown to be statistically significant when compared to retention by the uncoated membrane alone (ANOVA, Tukey; *p* < 0.0001) for all pH values tested.

Retention performance was then evaluated for MS2; a single-stranded RNA nonenveloped virus, with a diameter of approximately 26 nm and a p*I* of 3.9, and widely accepted as a nonpathogenic virus surrogate for poliovirus and as a virus challenge particle.^47,48^ Feed water containing 10^7^ plaque forming units per milliliter (pfu/mL) was pH adjusted to pH’s of 5, 7, and 9 and flowed through stacks of ten uncoated and ND-coated membrane discs (14 mm diameter, 4.3 mm μm total thickness, 0.66 cm^3^ total volume) under applied pressures between 0.25 and 0.5 bar. The permeate was assayed through methods outlined in the Methods and Materials section, and membrane retention was calculated in terms of its log_10_ reduction value (LRV), given by eq 2, where *N*_f_ is the number of viable viruses in the feedwater and *N*_p_ is the number of viable viruses in the permeate. As 100 μL samples were taken from the permeate and feed waters for analysis, the lower limit of detection is equal to 1 log_10_ of phage.
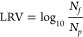
2Retention data for the MS2 bacteriophage is displayed in Figure 8. The uncoated membranes show very poor retention of the bacteriophage, less than 0.5 LRV across all measured volumes and pH values. Conversely, the nanodiamond-coated membranes display 6.2 LRV (>99.9999%) at all three pH values measured, though the LRV declines from 6.2 LRV to 2.4 LRV for large volumes of pH 9 feedwater passing through the ND-coated membrane stack. Since retention is proportional to the magnitude of the electrostatic interaction that leads to adsorption, the decline in retention is attributed to a lower membrane zeta potential at higher pH values, described in the zeta potential results of Figure 2a, which limits the total retention capacity of the membrane. For instances where the upper detection limit was reached, the membrane’s true LRV is still unknown since no bacteriophage were observed and counted on the assay plates, and therefore the LRV may well be far greater still.

Statistical analysis of the data sets was conductedwith GraphPad PRISM (version 8.1.1) using a two-way Analysis of Variance (ANOVA) with posthoc Tukey Test. The nanodiamond-coated membranes were found to be significantly more effective at retaining MS2 bacteriophage (ANOVA, Tukey; *p* < 0.0001) than the quartz membrane alone for all pH values tested.

## Conclusions

We have described a fabrication pathway that allows the extreme electropositive properties of hydrogenated detonation nanodiamond to be utilized for the filtration of viruses and other negatively charged contaminants found in drinking water. Nanodiamond was loaded onto quartz microfiber membranes to roughly 3.4% by mass, producing a near 4-fold increase in surface area from 23 to 88 m^2^/g. The membranes exhibited zeta potential values of +45 mV at pH 7 and an isoelectric point around pH 11, one of the highest zeta potentials ever reported in the literature for a separation platform. They showed excellent retention of the electronegative dye molecule acid black 2, and when challenged with MS2 bacteriophage exhibited LRV of at least 6.2, between pH 5 and 9. The combination of extreme positive zeta potential coupled with the apparent stability of the ND coating is very promising. Future work will now seek to confirm the ability to reuse the nanodiamond membranes by elution of the virus and other adsorbates from the membrane fibers. For virus capture and concentration in a lab setting or simply to remove filtered material from the membrane ready for reuse in drinking water processing, a number of different eluting solutions have previously found use. Typically, they exhibit an exceptionally high or low pH, so as to switch membrane or adsorbate surface charge.^30,49^ Since both quartz and diamond exhibit strong chemical stability, its anticipated that a wide range of eluting solutions could be effective. Another potential method of adsorbate removal is thermal treatment. Owing to the high thermal stabilities of nanodiamond and quartz, adsorbates may be burned off or denatured and flushed out if necessary. Furthermore, we note the possibility to regenerate the membrane’s positive surface charge surface by rehydrogenation of the nanodiamond itself, although this may only be economically feasible in a laboratory setting or on a large industrial scale. We also look to wider applications in the filtration of components of the blood, and into water and air sensing, since the functionisability of the nanodiamond surface makes the ND-coated membrane so versatile. Principally, we hope to develop the membrane into a universal low-cost and low-power input separation platform capable of producing clean drinking water that is accessible to all.

## Methods and Materials

### Synthesis of H-Terminated Detonation Nanodiamond Colloids

Detonation nanodiamond powder “purified grade 01” (PlasmaChem, Gmbh.) was placed into a vacuum tube furnace and the vacuum chamber evacuated to a base pressure better than 1 × 10^–5^ mbar. Hydrogen gas was flowed through the vacuum chamber at 100 sccm and the pressure maintained at 10 mbar. The vacuum chamber was resistively heated to 600 °C for 5 h, whereafter it was allowed to cool to room temperature under hydrogen gas flow. H-terminated nanodiamond powder (0.25 g) was dispersed in deionized water (500 mL) using a Sonics Vibra-cell VCX 500 ultrasonic processor. A duty cycle of 3 s on/2 s off was maintained for 6 h, under aggressive liquid cooling. The dispersion was allowed to settle overnight before being centrifuged using a Sigma 3–30 KS centrifuge at 40 000 g for 1 h, to remove nanodiamond aggregates. DLS particle size and zeta potential measurements of the resulting colloid were taken with a Malvern Zetasizer Nano ZS equipped with a 633 nm laser and a 173° backscattering angle. Particle size distributions were the result of 3 × 100 scans and typically gave an average (by number) particle size of 5 nm. An average zeta potential of +55 mV was usually observed, being the result of 3 × 100 zeta scans. The concentration of nanodiamond was determined by total evaporation of water from the nanodiamond colloid using a heat source at 100 °C, followed by gravimetric analysis of the remaining solid. An average nanodiamond concentration of 0.15 mg mL^–1^ was determined from five repeat measurements. To produce the various concentrations of nanodiamond colloid used in membrane fabrication, water was evaporated from the colloids under a controlled evaporation, using a heat source at 100 °C.

### Membrane Fabrication

Quartz microfiber membranes (Merck & Co. Inc.) were immersed in a bath of pristine colloidal nanodiamond for 3 h. Nanodiamond-coated membranes were removed from the nanodiamond bath and dried under atmospheric conditions overnight. The membranes were placed into a vacuum tube furnace and the vacuum chamber evacuated to a base pressure better than 1 × 10^–5^ mbar. The vacuum chamber was then resistively heated to 500 °C for 2 h, whereafter it was allowed cooled to room temperature. Once cool, membranes were continuously washed with deionized water to remove any nanodiamond not strongly bound to the fiber surface.

### Scanning Electron Microscopy

Morphology of quartz microfiber membranes prior to nanodiamond coating were characterized by SEM, using a Raith e-LiNE scanning electron microscope, operating at 20 kV and a 10 mm working distance.

### Transmission Electron Microscopy

Morphology of the quartz microfiber membranes prior to and following nanodiamond coating were characterized by TEM, using a JEOL JEM-2100 electron microscope, operating at 200 kV. Membrane samples were carefully broken apart and supported on a 3.05 mm copper mesh grid.

### Zeta Potential Characterization

Membrane zeta potential was calculated from streaming potential measurements using a SurPASS 3 electrokinetic analyzer. The SurPASS 3 comprises two Ag/AgCl electrodes at either end of a streaming channel, and the streaming potential is determined by measuring the potential difference between the inlet and outlet electrode, as a function of the electrolyte pressure. Disks of 14 mm membrane were mounted across the streaming channel, between two perforated support disks, and the permeability was controlled by compression of the membrane material. A 1 × 10^–3^ M solution of KCl was used as the electrolyte and driven through the membrane at pressures between 200 mbar and 600 mbar. The zeta potential was measured as a function of the electrolyte pH; 0.1 M HCl and 0.02 M NaOH solutions were used to induce pH change, using the SurPASS’s inbuilt titration system. Four measurements were taken at each pH value, an average was taken of the four measurements, and the resulting data points were plotted.

### BET Specific Surface Area

BET specific surface area was measured using a Quantachrome QuadraSorb-evo. Prior to BET analysis samples were degassed in situ at 150 °C for 2 h.

### Thermogravimetric Analysis

The extent of the nanodiamond coating was determined by thermogravimetric analysis (TGA). Measurements were taken using a PerkinElmer TGA4000 Thermogravimetric Analyzer. Samples were loaded into a crucible and heated between 30 and 900 °C at a heating rate of 5 °C min^–1^, under an air gas flow of 50 mL min^–1^. A detonation nanodiamond powder sample was analyzed under TGA to determine the temperature range over which it was oxidatively etched. The etch range was determined to begin when the weight (%) of the sample dropped below its lowest point following evaporation of water from the sample (at around 110 °C) and finish once the weight of the nanodiamond sample had dropped below 1% of its initial weight. Membrane samples were then evaluated under TGA across this same temperature range. The drop in weight of the uncoated quartz microfiber membrane across the temperature range of the oxidative etch was measured and subtracted from weight drops observed for nanodiamond-coated membranes, to derive the true “ND content” values that were then plotted.

### Retention Performance versus Acid Black 2

A total of 10 mg of acid black 2 powder (Merck & Co. Inc.) was dispersed in 1 L of deionized water, using an ultrasonic bath to ensure full dispersion. Acid black 2 containing feedwater was flowed through ten stacked membranes of cumulative thickness 0.43 cm, diameter 1.4 cm, and total volume: 0.66 cm^3^. Two perforated support disks held the membrane stack in place. Pressures between 250 mbar and 500 mbar were applied across the membrane housing to encourage feedwater flow. Ultraviolet–visible absorption measurements were taken of the feedwater and permeate, using the UV–vis Spectrophotometer GENESYS 10S, and acid black 2 concentrations were determined based on the absorption maximum for the dye molecule at 575 nm. Retention was calculated as the percentage of the dye molecule in the feedwater that was not present in the permeate, as described in the Results section.

### Retention Performance versus MS2 Bacteriophage

MS2 bacteriophage ATCC 15597-B1 was propagated at 37 °C overnight in 10 mL of sterile tryptone soya broth with the host bacterium *Escherichia coli* ATCC 15597. The broth suspension was centrifuged at 3000*g* for 10 min, and thereafter the supernatant was filtered by passing through a 0.2 μm cellulose acetate filter. The phage suspension was added to a 1 × 10^–3^ M NaCl solution to obtain phage concentrations on the order of 10^7^ plaque-forming units per milliliter (pfu/mL). To induce pH changes in the feedwater, 0.1 M NaOH and 0.1 M HCl solutions were used. MS2 containing feedwater was flowed through ten stacked membranes of cumulative thickness 0.43 cm, diameter 1.4 cm, and total volume 0.66 cm^3^. Two perforated support disks held the membrane stack in place. Pressures between 0.25 and 0.5 bar were applied across the membrane housing to encourage feedwater flow. 100 μL samples were taken from the feedwater and permeate for analysis. Dilutions of the samples were first prepared by adding SM buffer (an aqueous mixture of NaCl, MgSO4·7H2O, and Tris-Cl). Then 100 μL of diluted phage sample and 100 μL of the host bacterial strain *E. coli* ATCC 15597 (at a concentration of 5 × 10^7^ cfu/mL) were added to 5 mL of 65% molten nutrient agar containing 500 μM CaCl. The nutrient agar was maintained at a temperature of 45 °C by water bath, then poured over a tryptone soya agar plate. After 30 min resting time at room temperature, the plates were inverted and placed in an incubator at 37 °C for 16–20 h. The pfu were counted and compared with the feed assay to determine the retention in terms of the log reduction value. As 100 μL samples were used, the lower limit of detection is equal to 1 log of phage. The statistical significance of data sets was evaluated with GraphPad PRISM (version 8.1.1) using a two-way Analysis of Variance (ANOVA) with posthoc Tukey Test. All experiments were performed in three independent biological replicates.
